# A study on association of virulence determinants of verotoxic *Escherichia coli* isolated from cattle calves

**DOI:** 10.14202/vetworld.2016.915-918

**Published:** 2016-08-29

**Authors:** Singh Parul, Basanti Bist, Barkha Sharma, Udit Jain, Janardan K. Yadav

**Affiliations:** 1 State Veterinary Hospital, Dhana Teja, Mathura, Uttar Pradesh, India; 2 Department of Veterinary Public Health, College of Veterinary Sciences and Animal Husbandry, UP Pandit Deen Dayal Upadhyaya Veterinary Science University, Mathura, Uttar Pradesh, India; 3 Department of Epidemiology and Preventive Veterinary Medicine, College of Veterinary Sciences and Animal Husbandry, UP Pandit Deen Dayal Upadhyaya Veterinary Science University, Mathura, Uttar Pradesh, India

**Keywords:** enterohemolysin gene (*hly*A), enterohemolysin toxin, intimin gene (*eae*A), verotoxic *Escherichia coli*, verotoxin genes (*vt1* and *vt2*), virulence determinants

## Abstract

**Aim:**

The present study was conducted to find the association among virulence determinants of verotoxic *Escherichia coli* (VTEC) isolated from cattle calf feces.

**Materials and Methods:**

A total of 216 cattle calf fecal samples were collected aseptically and processed under required conditions for the isolation of *E. coli*. The isolates were further subjected to multiplex polymerase chain reaction (mPCR) for the detection of virulent genes. All the VTEC isolates were serotyped at the Central Research Institute, Kasauli, Himachal Pradesh. The VTEC isolates were observed for the enterohemolysin production on washed sheep blood agar (wSBA).

**Results:**

A total of 177 presumptive *E. coli* were isolated from 216 calf fecal samples revealing an overall prevalence of *E. coli* to be 81.94%. A total of 32 (14.81%) isolates were detected as VTEC through mPCR. The prevalence of verotoxin genes *vt1*, *vt2*, and combination of *vt1*+*vt2* in the VTEC isolates was found to be 12 (37.5%), 14 (43.75%), and 6 (18.75%), respectively. Other virulent genes *eae*A and *hly*A were found in 6 and 11 VTEC strains with prevalence values of 18.75% and 34.37%, respectively. A total of 13 different O serogroups were revealed in serotyping of 32 VTEC isolates. Out of 32 VTEC strains, only 26 (81.25%) were enterohemolytic on wSBA as they produced the characteristic small, turbid zone of hemolysis around the streaking line. Although enterohemolysin production has been attributed to the presence of *hly*A gene, only 11 of 26 enterohemolysin producing VTEC were found to be harboring the *hly*A gene (11/26) 42.03%.

**Conclusion:**

The present study concludes that there might be an association between the presence of verotoxin genes and enterohemolysin production in VTEC group of *E. coli*.

## Introduction

The emergence of verotoxin-producing *Escherichia coli* (VTEC) is one of the biggest challenges for food safety and cattle industry worldwide [1]. The VTEC are also known as Shiga toxin-producing *E. coli* and are responsible for producing a spectrum of illness in humans including diarrhea, hemorrhagic colitis, hemolytic uremic syndrome (HUS), and thrombotic thrombocytopenic purpura [2,3]. Most of the VTEC associated foodborne infections in humans are supposed to be consumption of foods (undercooked ground beef, unpasteurized dairy products, and vegetables) and water contaminated with ruminant feces and other environmental sources [4,5].

The ruminants are established natural reservoirs of VTEC, carrying it asymptomatically in their intestine and excreting in feces [6]. The prevalence of the VTEC in the cattle feces is generally reported to be high especially in calves [7,8]. More than 400 O:H serotypes of VTEC have been isolated from cattle and humans while over 100 serotypes are found to be linked with human diseases [9,10]. The pathogenicity of VTEC is attributed to a number of virulence factors, including potent verotoxins (VT1 and VT2, encoded by phage-mediated genes *vt1* and *vt2*), an intimin protein associated with attaching and effacing lesions (encoded by *eae*A gene) and enterohemolysin, a lytic toxin (encoded by *hly*A gene) [11-13]. Several studies have indicated the association between these virulence factors such as *vts*, *hly*A genes, and enterohemolysin production in VTEC serotypes originating from varied sources [14,15], but more research is needed to prove their association.

Thus, this study aims to find the association in virulence determinants of VTEC isolated from cattle calf feces.

## Materials and Methods

### Ethical approval

For this study faecal samples were collected from immediately voided faeces of animals. So this work does not require any ethical approval.

### Sampling, enrichment, and phenotypic characterization of isolates

A total of 216 fecal samples of cattle calves (below 1 year) were collected from three organized dairy farms of Uttar Pradesh state, from June 2012 to May 2013. All the samples were transported under chilled conditions and processed immediately in the laboratory for the better recovery of *E. coli*. The 1 g of feces was enriched in 10 ml of trypticase soya broth (HiMedia, India) containing acriflavine (10 mg/L) and incubated at 37°C for 24 h [16]. The enriched inoculum was streaked on MacConkey agar and the resulting lactose fermenting pink, smooth, and round colonies were further streaked on the selective eosin methylene blue (EMB) agar (HiMedia, India) and incubated at 37°C for 24 h. The colonies showing green metallic sheen on EMB agar were picked as presumptive *E. coli* and streaked on nutrient agar slants and biochemically confirmed by kit (KB010 Hi *E. coli* Identification kit-HiMedia, India).

### Virulence detection

A multiplex polymerase chain reaction (mPCR) was carried out using four sets of oligonucleotide primers for detection of virulent genes *vt1, vt2, eae*A, and *hly*A [17]. The template DNA was extracted from a single colony of each isolate using kit (Genei, Bengaluru). The purity and concentration of extracted DNA were detected by nanodrop method (Eppendorf, German). The every 25 µl PCR reaction mixture of contained ×1 PCR buffer, 1.5 mM MgCl_2,_ each primer at a concentration of 40 nM, 200 µM each of deoxynucleotide triphosphates, one unit of Taq DNA polymerase, and 2.0 µl of template DNA. The PCR reaction was performed in a thermal cycler (Cyber Lab, India) using standard cycling conditions and amplified products were separated by electrophoresis on 2% agarose gel and visualized with ethidium bromide. Standard 100 bp DNA ladder (Genei, Bengaluru) was used as marker.

### Serotyping and detection of enterohemolysin (E-hly) toxin

All the isolates that turned out to be positive through mPCR method were also serotyped at the National Salmonella and *Escherichia* Center, Central Research Institute, Kasauli, Himachal Pradesh, India.

Enterohemolysin production in VTEC isolates was observed as per the method of Beutin *et al*. [18]. All the isolated VTEC were streaked over 5% washed sheep blood agar (wSBA) plates supplemented with 10 mM calcium chloride incubated at 37°C and examined at 18 h. The VTEC isolates producing small turbid hemolytic zone around the streaking line after 18 h were considered as enterohemolytic.

## Results

### Phenotypic characterization of VTEC

A total of 177 presumptive *E. coli* were isolated from 216 fecal samples revealing a total prevalence of 81.94%. These isolates showed pink-colored smooth colonies on MLA and produced distinct, clear greenish metallic sheen over EMB. All the isolates showing following biochemical reactions as negative for Voges-Proskauer, hydrogen sulfide, citrate, oxidase and urease production, and positive for indole and methyl red tests were biochemically confirmed as *E. coli*.

### Detection of virulent genes

A total of 32 *E. coli* isolates from calf feces found to be positive for virulent genes (*vt1, vt2, eae*A, and *hly*A) by mPCR showing a prevalence of 14.8%. The virulent genes were present either singly or in combinations, showing a prevalence of *vt1*, *vt2*, and (*vt1*+*vt2*) genes as 12 (37.5%), 14 (43.75%), and 6 (18.75%), respectively. Other virulent genes *eae*A and *hly*A were present in 6 and 11 VTEC strains showing prevalence values 18.75% and 34.37%, respectively (Table-1).

**Table-1:** Distribution of virulent genes (*vt1, vt2, eae*A, and *hly*A) and enterohemolysin production in VTEC isolates from cattle calves feces.

Serotypes	Number of VTEC isolates	*vt1*	*vt2*	*vt1+vt2*	*eae*A	*hly*A	Enterohemolysin production
O9	4	−	−	+	−	+	+
O11	5	+	−	−	−	+	+
O26	1	+	−	−	+	−	+
O27	2	−	+	−	−	−	+
O34	2	−	+	−	−	−	−
O52	4	−	+	−	−	−	−
O56	3	−	+	−	−	−	+
O81	3	+	−	−	+	−	+
O83	2	+	−	−	−	−	+
O84	1	−	+	−	−	−	+
O91	1	+	−	−	+	−	+
O134	2	−	+	−	−	−	+
O156	2	−	−	+	+	+	+

VTEC=Verotoxic *Escherichia coli*

### Serotyping

A total of 13 different O serogroups were revealed on serotyping of 32 VTEC isolates (Table-1). Of these serotypes, four were identified as O9, O26, O91, and O156, which have been shown to be associated with HUS in human being.

### Detection of enterohemolysin (E-hly) in VTEC

In the hemolysis assay, out of 32 VTEC isolates, 26 (81.25%) showed the enterohemolysin production on wSBA as they produced small, turbid zone of hemolysis around the streaking line. All the 26 enterohemolytic VTEC isolates revealed with verotoxin genes as 12 (46.5%) had *vt1* gene, 8 (30.76%) isolates harbored *vt2* gene, and 6 (23.07%) isolates had both the genes (*vt1+vt2*). Although enterohemolysin production has been attributed the presence of *hly*A gene, only 11 of 26 enterohemolysin producing VTEC were found to be harboring the *hly*A gene 11 (42.03%).

## Discussion

In the present study, the prevalence of *E. coli* in the feces of cattle calves was 81.94%. The prevalence value of current study had nearly coincided with the findings of other countries as in Iran (86.7%) [19], Egypt (75.6%) [20], and India (75%) [21], whereas the lower prevalence was reported in Egypt (63.6%) [22].

During the study, 14.81% *E. coli* isolates were found as VTEC through mPCR in the calves feces. The serotyping results of the current study depicted that all the VTEC isolates were belonged to the non-O157 VTEC group. The wide range of prevalence values of non-O157 VTEC (0.42 to 74%) and *E. coli* O157:H7 (0.2 to 48.8%) has been reported from cattle feces worldwide [23]. The prevalence of VTEC reported in our study lies within globally reported a range of VTEC from cattle feces. Almost similar prevalence of VTEC has been reported in another study from India by Mishra *et al*. 13.4% [24] while higher prevalence values have been reported from other places such as Egypt (26.7%) [25], California (37.9%) [26], Ireland (40%) [27], and Florida (58.1%) [6]. The wide variations in prevalence values of *E. coli* and VTEC in calves feces can be attributed to epidemiological determinants such as stocking density, age, sex, and season, whereas sampling strategy, sample handling, and laboratory methods might also have profound effect on prevalence values reported in different countries [28,29].

The enterohemolysin is a lytic toxin which is encoded by a large virulence plasmid and was initially supposed to be produced by VTEC group of *E. coli*. The association of enterohemolysin production and VTEC group of *E. coli* was first identified by Beutin *et al*. [18] and later on this correlation was reported by numerous other research workers. In this investigation, the prevalence of enterohemolysin production was 26 (81.25%) which is almost similar to 89.9% reported by Rathore *et al*. [30] from cattle feces. In various other studies, prevalence values of enterohemolysin production by VTEC isolated from feces and other sources have been found as 31.18% [15], 68.29% [31], 86% [14], and 97.6% [32].

In few studies, a genomic relation has been studied with the production of enterohemolysin toxin in VTEC group of *E. coli*. In a study by Meng *et al*. [33], all 120 *E. coli* O157:H7 isolates harboring verotoxin genes produced the enterohemolysin. In another report, all the *E. coli* O157 isolates were found positive for *hly*A gene and enterohemolytic phenotype [34]. In spite of the association of enterohemolysin production with *hly*A gene, the verotoxin gene *vt1* was also found evident in enterohemolysin production in non-O157 VTEC isolates [15]. In the current study, the 26 non-O157 VTEC isolates were enterohemolytic on wSBA and only 11 VTEC isolates (40.03%) were revealed the presence of *hly*A gene while the rest of isolates were harbored either *vt1, vt2*, or both (*vt1+vt2*) the verotoxin genes but still produced this toxin on wSBA (Table-1). It enforces the fact that the mere presence of *hly*A gene might not be the sole factor for the production of enterohemolysin, but the presence of verotoxin genes (either *vt1*, *vt2*, or both) also might favor its production of enterohemolysin thereby enhance the virulence of VTEC. The present study concludes that there might be an association between the presence of verotoxin genes and enterohemolysin production in VTEC group of *E. coli*.

## Conclusion

The zoonotic agent VTEC contaminates the food chain and constitutes the major public health hazard. It is necessary to know all possible virulence factors for its control. The present study concludes that there might be an association between the presence of verotoxin genes and enterohemolysin production in VTEC group of *E. coli*. There is further need to study more on virulence factors of VTEC.

## Authors’ Contributions

SP, BB, BS, UJ, and JKY executed the study design and analyzed the data. SP performed the entire study under the guidance of BB. SP drafted and revised the manuscript with the help of BS and UJ. All authors read and approved the final manuscript.
